# Draft Genome Sequences of Three Virulent Streptococcus thermophilus Bacteriophages Isolated from the Dairy Environment in the Veneto Region of Italy

**DOI:** 10.1128/genomeA.00045-18

**Published:** 2018-03-08

**Authors:** Vinícius da Silva Duarte, Sabrina Giaretta, Laura Treu, Stefano Campanaro, Pedro M. Pereira Vidigal, Armin Tarrah, Alessio Giacomini, Viviana Corich

**Affiliations:** a Department of Microbiology, Federal University of Viçosa, Viçosa, Minas Gerais, Brazil; b Department of Agronomy, Food, Natural Resources, Animals and the Environment, University of Padova, Legnaro, Italy; c Department of Biology, University of Padova, Padua, Italy; d Department of Environmental Engineering, Technical University of Denmark, Lyngby, Denmark; e Núcleo de Análise de Biomoléculas (NuBioMol), Center of Biological Sciences, Federal University of Viçosa, Viçosa, Minas Gerais, Brazil

## Abstract

Streptococcus thermophilus, a very important dairy species, is constantly threatened by phage infection. We report the genome sequences of three S. thermophilus bacteriophages isolated from a dairy environment in the Veneto region of Italy. These sequences will be used for the development of new strategies to detect and control phages in dairy environments.

## GENOME ANNOUNCEMENT

Streptococcus thermophilus is a low-GC Gram-positive bacterium considered the second most important dairy species (1) and is commonly used to produce cheese and yogurts (2, 3). Currently, its statuses of generally recognized as safe (GRAS) and of qualified presumption of safety (QPS) (4) make it reach a market value of $40 billion (5, 6).

Ubiquitous in the dairy environment, bacterial viruses or bacteriophages are a constant threat to S. thermophilus starter cultures (7, 8). Overall, economic losses due to phage infection in dairy products are related to low fermentation activity and reduced product quality that may lead to total process failure (9, 10).

Here, we report the genome sequences of three S. thermophilus bacteriophages isolated from a dairy environment in Northeast Italy, vB_SthS_VA214, vB_SthS_VA460, and the partial genome sequence of vB_SthS_VA698 (VA214, VA460, and VA698, respectively).

Bacteriophages were concentrated and purified using polyethylene glycol 8,000, and their genomic DNA was extracted following the method described by Binetti et al. (11). Sequencing was performed with the Illumina MiSeq platform using paired-end (PE) reads (2 × 250 bp) and a Nextera library at the Ramaciotti Centre for Genomics (Sydney, Australia). After quality filtering and merging of the overlapping PE reads, a total of 56,194, 57,208, and 68,210 sequences were obtained. Raw reads were assembled *de novo* using CLC Genomic Workbench software (version 9.5). Coverage values obtained for VA214, VA460, and VA698 were approximately 367-, 308-, and 122-fold, respectively. Total genomes sizes of 38.2, 41.2, and 33.3 Kb were estimated for VA214, VA460, and VA698, respectively, with an average GC content of 38.6%.

The Rapid Annotations using Subsystems Technology (RAST) server (12) was used for gene finding and annotation. In total, 53, 56, and 38 coding sequences (CDS) were predicted for VA214, VA460, and VA698, respectively. For phage VA214 only, a gene cluster encoding seven tRNAs (Gly, Ala, Val, Lys, Leu, Thr, and Gly) and without introns or pseudogenes was identified using the tRNAscan-SE program (13).

A BLASTn similarity search revealed that the genome of VA214 is related to Streptococcus phage P0091, whereas VA460 and VA698 are closely related to Streptococcus phage P9851 and Streptococcus phage P7132, respectively.

In this work, we present the complete genome sequences of three S. thermophilus bacteriophages, which will serve as the basis for developing new strategies useful to detect and control phages in dairy environments. In the future, we will also conduct studies of bacterial immunity against phages using S. thermophilus M17PTZA496 (14) and S. thermophilus TH1435/1436 (15).

### Accession number(s).

This whole-genome shotgun project has been deposited at DDBJ/ENA/GenBank under the accession numbers MG708274, MG708275, and MG708273 for vB_SthS_VA214, vB_SthS_VA460, and vB_SthS_VA698, respectively. The versions described in this paper are the first versions, MG708274.1, MG708275.1, and MG708273.1, respectively.
